# The effectiveness of unsupervised home-based exercise for improving lower extremity physical function in older adults in Western and Eastern cultures: a systematic review and meta-analysis

**DOI:** 10.1186/s12877-024-05393-4

**Published:** 2024-10-01

**Authors:** Ian Ju Liang, Oliver J. Perkin, Polly M. McGuigan, Bruno Spellanzon, Molly Robb, Chien-Yu Liu, Linda L. Lin, Dylan Thompson, Max J. Western

**Affiliations:** 1 Department for Health, University of Bath, 1 West 5.108, Bath, BA2 7AY UK; 2 Centre for Nutrition, Exercise and Metabolism, University of Bath, Bath, UK; 3 Department of Physical Education and Sport Sciences, National Taiwan Normal University, Taipei, Taiwan; 4 Graduate Institute of Physical Education, Health & Leisure Studies, National Cheng Kung University, Tainan, Taiwan; 5 Centre for Motivation and Health Behaviour Change, University of Bath, Bath, UK

**Keywords:** Unsupervised exercise intervention, Home-based activities, Lower extremity physical performance, Ageing population, Independent living

## Abstract

**Background:**

Ageing leads to decreased physical function, which can impact independent living and raise health risks, increasing demand on healthcare resources. Finding affordable and accessible exercise to improve physical function is necessary for a population seemingly resistant to strength and balance training in leisure settings. This review aimed to evaluate whether unsupervised home-based exercises improve lower extremity function in older adults.

**Methods:**

We systematically searched for randomised controlled trials (RCTs) and cluster RCTs investigating unsupervised home-based exercises’ effects on physical function in older adults through English and Mandarin databases. Studies’ methodological quality was assessed using the Cochrane’s Risk of Bias Tool. Meta-analyses were conducted on lower extremity functions outcomes.

**Results:**

Of the 6791 identified articles, 10 English studies (907 participants) were included, 8 studies (839 participants) were used for final meta-analysis, with no Mandarin studies. Studies were largely based in Europe with mostly moderate risk of bias. Most interventions were multicomponent lasting 10–40 min/session, 3 times/week. Meta-analysis showed no statistically significant differences in 5 sit-to-stand (*p* = 0.05; I^2^ = 0%), maximal knee extension strength (*p* = 0.61; I^2^ = 71%), 10 m maximal walking speed (*p* = 0.22; I^2^ = 30%), timed-up-to-go (*p* = 0.54; I^2^ = 0%), and short physical performance battery (*p* = 0.32; I^2^ = 98%) between exercise and control groups.

**Conclusions:**

This meta-analysis suggests that unsupervised home-based exercise programmes have little impact on lower extremity functions in older adults. This review is limited by the small number of included studies, sample sizes, and high heterogeneity. There is a need to understand why this format lacks efficacy, and design more beneficial home-based exercise programmes.

**Supplementary Information:**

The online version contains supplementary material available at 10.1186/s12877-024-05393-4.

## Background

The number of people over the age of 65 is expected to more than double by 2050 [1]. Specifically, according to the World Health Organization, the number of older adults in the European region and Asia region are forecast to increase by 11% and 12.3% from 2010 to 2050 respectively. Ageing is associated with physiological deterioration, for example muscle function decline [2–4], mobility and functional balance decline [5–7], and an increased risk of osteoporosis and/or sarcopenia caused by physical inactivity [8–11]. These physical declines can lead to higher risk of falls and hospitalisation, and ultimately loss of independence. As societies become ‘super-aged’ (with over 20% of total populations aged over 65) [12], prevalence of health related issues are predicted to rise, impacting quality of life and straining healthcare resources [13, 14]. Interventions that support adults to maintain robust physical function in later life are essential for improving wellbeing and minimising the economic burden of physical frailty and associated morbidity [15, 16].

Regular physical exercise is recommended to prevent a loss of physical function, maintain activities of daily living, and decrease disability in older adults [17–19]. Research has explored the effect of exercise on physical fitness, balance, falls, and well-being in older adults, revealing that progressive resistance training significantly improves muscle strength and performance [20]. The trainer-led ‘Otago’ exercise programme for example, focusing on leg strength and balance, has been shown to improve mobility, balance, and muscle strength, and reduced falling risk and mortality [21–23]. Multicomponent exercises, including strength, endurance, and balance training, effectively maintained or even improved lower limb physical function in older adults [24], especially in those who are diagnosed as frail [25, 26]. In fact, even endurance exercise alone, which would not typically be expected to improve strength related outcomes, enhanced muscle functions in older adults with minimal strength [27, 28]. Accordingly, functional/strength improvements might be a biproduct of any exercise programmes in older populations. It is clear that all forms of exercise bring benefits in physical fitness in older adults with different physical activity levels.

It is commonly reported that older adults experience specific barriers to exercise, such as self-reported lack of time, apathy towards exercise, lack of access or convenient spaces for activity, feeling pressured or a lack of belonging in group leisure or gym settings, lack of self-efficacy for exercise, and economic constraints [29–33]. These barriers can make exercise programmes in specialist exercise facilities unfeasible for many older adults, while supervised exercise provision, particularly non-group based, is a resource intensive form of intervention. Given these challenges, exploring alternatives to traditional exercise provision is necessary. Unsupervised, home-based exercise could be a viable, low-cost strategy for promoting physical activity for older adults. This approach may help to overcome typical participatory barriers, particularly when the format is no or low cost, motivating, safe, and easy to undergo in one’s familiar home environment [34–36]. Previous reviews found supervised and minimally supervised home-based exercises to be advantageous to muscle strength, mobility, and balance [37–39]. However, some studies have demonstrated that unsupervised home-based exercise interventions may have higher adherence rates, presenting a practical alternative to resource intensive supervised training programmes [40–42].

While evidence supports unsupervised home-based exercise feasibility and acceptability, its effectiveness has not yet been comprehensively reviewed. Additionally, a criticism of previous reviews is that they are limited to the monolingual scope of searches, potentially neglecting a large body of literature. This study aimed to fill this gap by exploring the efficacy of exclusive home-based exercise as a preventative, low-cost strategy for older adults and by identifying the optimal format of exercise that meets the needs of this population. Furthermore, considering cultural differences is crucial for suggesting appropriate exercise training methods to older adults from different countries, as exercise participation is affected by cultural values [43]. To our knowledge, there have been no attempts to synthesize the evidence on the cultural differences of the effective and optimal mode of home-based exercise interventions in older adults in one review. This study aimed to review the effectiveness of unsupervised, home-based exercises on lower extremity physical functions in older adults and investigate whether the effects and benefits of different unsupervised home-based exercises vary across Western and Eastern cultures.

## Methods

The study protocol was registered with the International Prospective Register of Systematic Reviews (PROSPERO) (registration number: CRD42023395942, available from: https://www.crd.york.ac.uk/prospero/display_record.php?ID=CRD42023395942). This systematic review followed the Population, Intervention, Comparison, Outcomes and Study (PICOS) framework to determine the design of the review search and extraction strategy. In summary, the *participants* of the study were community dwelling older adults from Western (namely anglospheric European countries) or Eastern (namely sinospheric east Asian countries) cultures. The *intervention* of interest was any self-directed strength balance or general physical function programme targeting the lower body. The *comparison* group were participants undertaking usual care or an active non-exercise intervention. The *outcomes* of interest were quantitative measures of strength, balance, or general physical function. And the *study* design was a randomised controlled trial. We followed PRISMA guidelines to ensure all relevant information was included (see Supplementary file 1).

### Eligibility criteria

Studies had to be: (a) RCTs or cluster RCTs; (b) self-guided home-based exercise interventions of any type; (c) control groups with usual care or no exercise; (d) objective quantitative outcome measures of strength and balance or general physical function; and (e) community dwelling or non-institutionalised older adults aged over 65 years who did not reside in nursing or care homes. No restriction on disease or cognitive function was included, however, studies whose participants had physical disability (i.e., wheelchair users, SCI patients) precluding lower-limb exercise were excluded. Studies were excluded if they were: (a) observational, quasi-experimental, single group designs, and feasibility or qualitative studies; (b) without intervention descriptions; (c) group-based, gym-based, and lab-based settings; (d) upper-body training only; (e) interventions with supervision, home visits, follow-up visits, or any in-person/telephone feedback for progression adjustment; (f) without strength- or balance-related outcome measures; and (g) non-English or non-Mandarin publications. Interventions needed to be self-guided; a one-off introduction session or non-instructive calls (i.e., motivational calls) were permissible.

### Search strategy

We included English and Mandarin literatures regarding the cultural context. The English studies included those being conducted in Europe, North America, Australia and New Zealand, whereas the Mandarin studies had to be conducted in East Asian states. English literature was searched on March 6, 2020, and updated on April 27, 2022, in PubMed, Web of Science, and Embase. Mandarin literature search dates were May 23, 2020, and June 12, 2022, in AiritiLibrary, China National Knowledge Infrastructure, and NCL Taiwan Periodical Literature databases.

The selection of keywords for the search was carefully designed with the guidance of an expert subject librarian, in collaboration with an experienced team of exercise and behavioural scientists. This interdisciplinary approach ensured that the search strategy was comprehensive and aligned with the objectives of the review. In addition, reference lists from relevant systematic reviews and meta-analyses were searched to identify any additional studies. Manual searches and literature extracts were conducted along with the screening reference lists of systematic reviews. See Supplementary file 2 for the searching strategy and terms.

### Identification of relevant studies

After database searches, five reviewers screened study titles and abstracts. Full texts were sought for articles not excluded after title and abstract screening. English study selection involved IJL, MR, and BS; Mandarin study selection involved IJL and CYL. Disagreements were resolved by MW and OP. Full texts were reviewed in two phases: phase one involved two reviewers discussing eligibility, and phase two involved single-author (IJL) screening. Unclear cases were discussed with MW. In the case of missing, incomplete, or unclear information, the corresponding authors were contacted to seek additional data or further details.

### Assessment of methodological quality

Methodological quality was evaluated using Cochrane’s Risk of Bias Tool 2.0 [44], which assesses six domains (randomisation process, deviations from interventions, missing data, outcome measurement, selection of the reported result, and overall bias). Domains were rated as “low risk of bias”, “some concerns”, or “high risk of bias”. A study was judged as low risk of bias if all domains were classified as low risk, some concerns if it had concerns in at least one domain but not to be at high risk of bias for any domain, and high risk if it had high risk of bias in any domain [44]. Three reviewers (IJL, MW and BS) independently assessed the studies’ quality. The certainty of the evidence was analysed using the Grading of Recommendations, Assessment, Development, and Evaluations (GRADE) framework [45].

### Data extraction

Data from each study were extracted by two reviewers (IJL and BS) independently. Descriptive data extracted included: authors, study country, participant characteristics (age, gender, sample size, health status, physical activity level), intervention delivery type (DVD, computer units, online platforms, with or without one-off introductory session), duration and frequency of interventions, intervention type, follow up period, and outcome measures.

### Data analysis

Meta-analyses were conducted to examine the effects on lower extremity functions. Hedges’s g calculated standardized mean differences between intervention and control arms [46]. A random effects model, using the restricted maximum likelihood method [47], was utilised to calculate the pooled effect size, as the included studies had different conditions (e.g. types of exercise, exercise intensity, intervention duration, target populations) which means the heterogeneity between the studies is assumed [46]. The I^2^ test for heterogeneity was used to identify the proportion of variability across studies [48]. SPSS version 28 (IBM Corp, Armonk, NY) was used for all analyses.

## Results

**Fig. 1:**
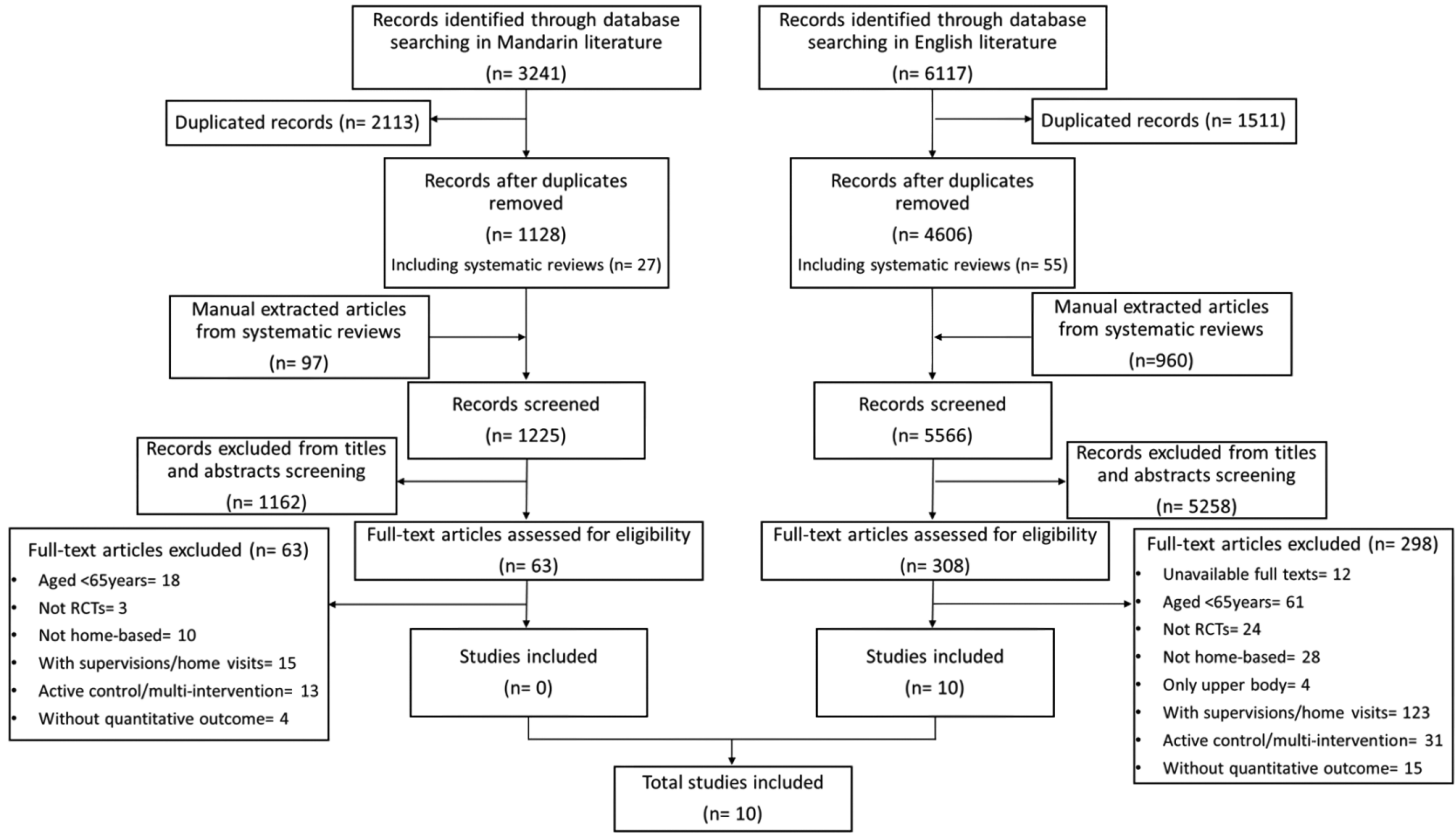
Literature screening flowchart

From the English database, 4606 records, including 55 systematic reviews, were identified after removing duplicates. There were 960 articles manually extracted from those 55 systematic reviews. This led to 5566 titles and abstracts screening, resulting in 308 articles for full-text screening. Accordingly, 298 articles were excluded, leaving 10 articles for inclusion in this systematic review. From the Mandarin database, 1128 records were returned, including 27 systematic reviews. 97 articles were extracted manually from those 27 systematic reviews. Thus, 1225 titles and abstracts were screened. Of these 1225 articles, 63 articles were included for full-text screening. Consequently, none of the articles were included. See Fig. 1. for the screening process.

### Study characteristics

The ten included studies were all RCTs, with three based in the USA [49–51], four in Europe [52–55], two in Australia [56, 57], and one in Asia (Japan) [58], which were all identified in the English published literature. Accordingly, comparisons between Western and Eastern cultures in terms of effectiveness were not possible, and the results from herein compiles all studies included in the review. See Supplementary file 3 for the summary of the individual study characteristics.

#### Participants

Six studies recruited community dwelling older adults [49, 50, 52, 54, 56, 57], one study [55] recruited participants from a rehabilitation centre, and the remaining three studies [51, 53, 58] recruited participants from clinical practices for special populations (i.e., patients with knee osteoarthritis, dialysis, and prostate cancer). Three studies recruited female participants only [54, 55, 58], and one involved male participants only [51]. In total, 907 participants were involved in these 10 studies, with 264 men, 611 women, and 32 with unreported sex or gender. Amongst them, 455 were in exercise groups and 452 in control groups.

#### Exercise interventions

Across the included 10 studies, there were 11 different home-based exercise programmes comprising multicomponent interventions focusing on balance, strength, mobility, and flexibility (*n* = 8) [49–51, 54–57] (specifically; balance, strength and flexibility [49, 54]; balance and mobility [56, 57]; strength and mobility [55]; balance, strength, and mobility [50, 51]), walking (*n* = 1) [53], stretching (*n* = 1) [58], and Tai-chi ‘inspired’ exercises (*n* = 1) [52]. Eight interventions had one-off introductory sessions (as described in Supplementary file 3). Delivery types varied, with two using DVD, four using computer/ tablet systems, four with written instructions, and one via online platforms (i.e., prerecorded YouTube videos). Interventions lasted 1 to 24 months, often 3 times weekly (*n* = 6), lasting 10 to 40 min per session.

#### Outcome measures

Regarding the instruments for lower extremity functional assessment, five studies assessed the 5 x sit-to-stand test and two assessed knee extension strength as markers of lower extremity strength; five studies measured 10-meter (m) walking speed and three measured time-up-to-go (TUG) for mobility [59]; and three studies evaluated overall lower extremity function with the Short Physical Performance Battery (SPPB) [60]. However, of the 10 included studies, only 8 studies, involving 839 participants, were finally used for statistical meta-analyses due to the lack of essential data (i.e., mean and standard deviation) of two studies [50, 52]. Thus, meta-analysis was conducted in the following outcome measures: 5 x sit-to-stand (*n* = 5) [53–57], knee extension strength (*n* = 2) [55, 57], 10 m walking speed (*n* = 4) [54–56, 58], TUG (*n* = 2) [56, 57], and SPPB (*n* = 3, including one three-arm study) [49, 51, 56]. Where one study [51] was a three-arm RCT with two exercise intervention arms and one control arm, the control group was split equally between 2 intervention arms to avoid double counting of participants.

##### The effectiveness on 5 x sit-to-stand time

**Fig. 2:**
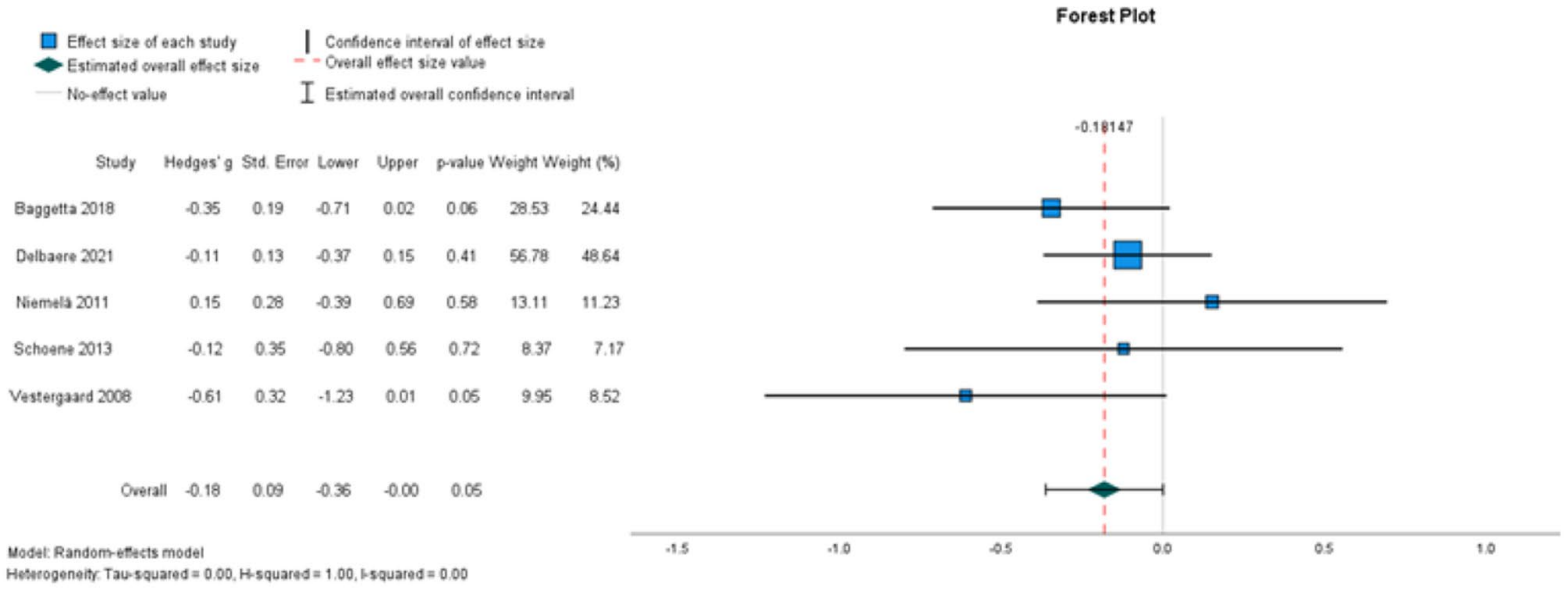
Forest plots depicting the pooled standardised mean difference in 5 x sit-to-stand time. Outcomes favouring intervention are depicted to the right, favouring shorter time performing 5-time sit-to-stand test

Five studies, involving 464 participants, were included for meta-analysis (Fig. 2). Pooled results demonstrated that the unsupervised home-based exercise interventions did not have a statistically significant intervention effect on time to complete 5 x sit-to-stands from a chair (random-effects estimate: -0.18, 95% CI [− 0.36,0.00]). Heterogeneity was low (I^2^ = 0%).

##### The effectiveness on knee extension strength

**Fig. 3:**
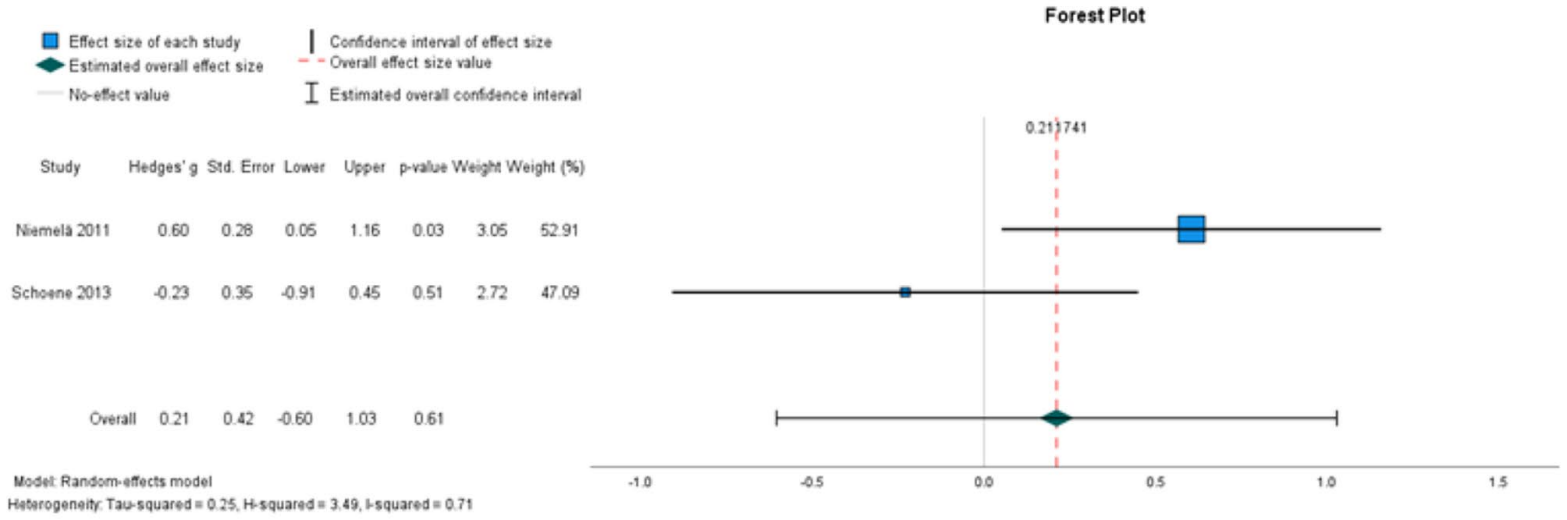
Forest plots depicting the pooled standardised mean difference in maximal knee extension strength. Outcomes favouring intervention are depicted to the left

Two studies, involving 83 participants, were included for meta-analysis (Fig. 3). The analysis did not identify a statistically significant intervention effect in knee extension strength (random-effects estimate: 0.21, 95% CI [− 0.60,1.03]). I^2^ = 71% represented substantial heterogeneity.

##### The effectiveness on 10 m walking speed

**Fig. 4:**
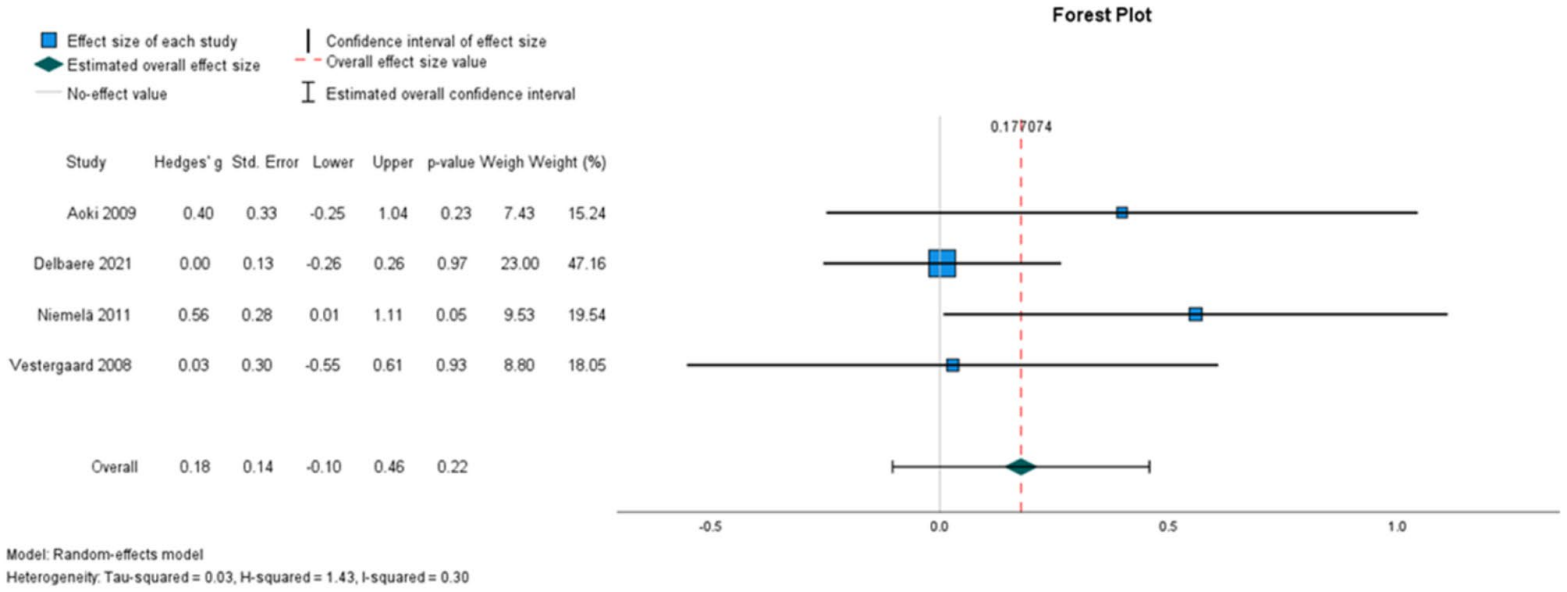
Forest plots depicting the pooled standardised mean difference in 10-metre maximal walking speed. Outcomes favouring intervention are depicted to the left

Four studies, involving 357 participants, were included for meta-analysis (Fig. 4). Pooled results revealed no statistically significant intervention effect in 10 m walking speed (random-effects estimate: 0.18, 95% CI [− 0.10,0.46]). Heterogeneity was moderate (I^2^ = 30%).

##### The effectiveness on timed-up-to-go (TUG)

**Fig. 5:**
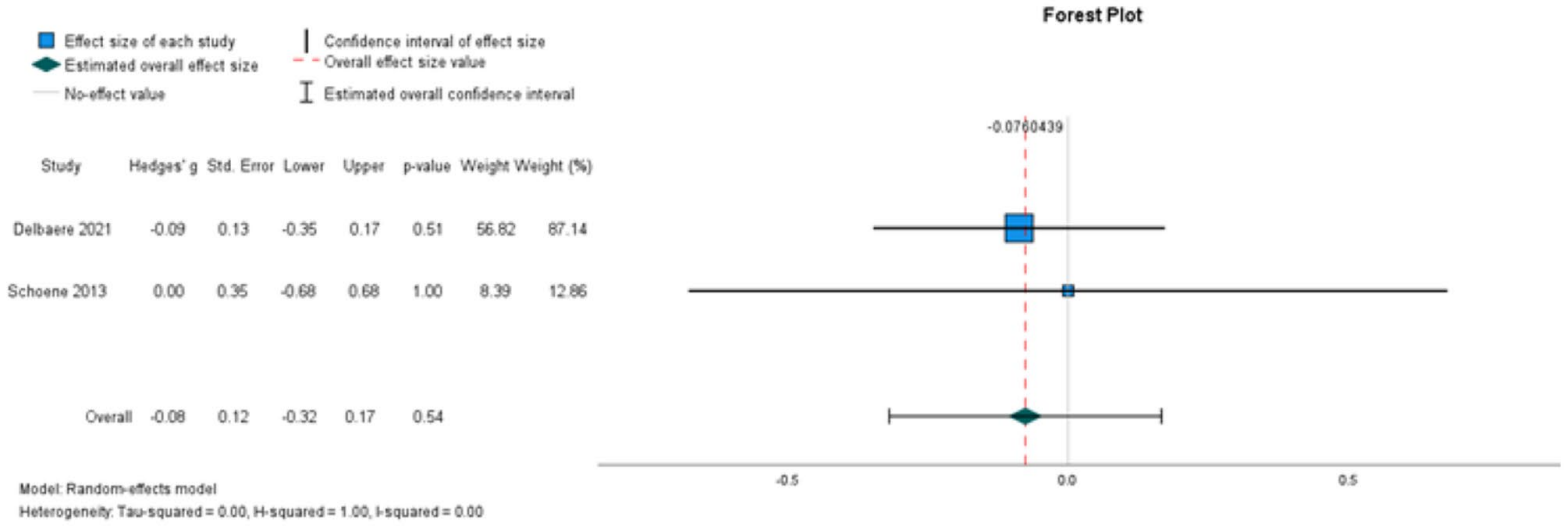
Forest plots depicting the pooled standardised mean difference in TUG performance. Outcomes favouring intervention are depicted to the right

Two studies, involving 258 participants, were included for meta-analysis (Fig. 5). The analysis did not identify a statistically significant intervention effect on TUG performance (random-effects estimate: -0.08, 95% CI [− 0.32,0.17). Heterogeneity was low (I^2^ = 0%).

##### The effectiveness on SPPB

**Fig. 6:**
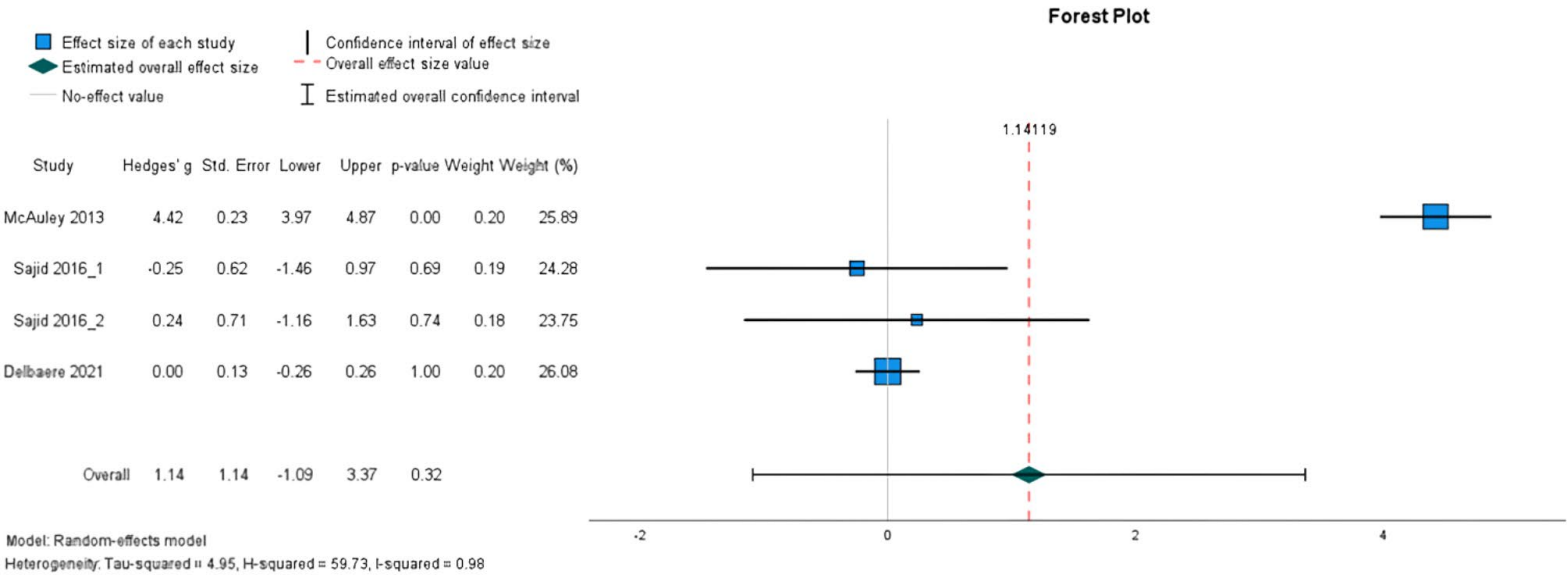
Forest plots depicting the pooled standardised mean difference in SPPB score. Outcomes favouring intervention are depicted to the left

Three studies (one study [51] appears twice as it was a three-arm trial), involving 505 participants in total, were included for meta-analysis (Fig. 6). No statistically significant intervention effect was observed on SPPB score (random-effects estimate: 1.14, 95% CI [− 1.09,3.37]). Heterogeneity was considerable (I^2^ = 98%).

Overall, the meta-analysis, using random-effects analysis, showed that there were no significant differences between unsupervised home-based exercise programmes and control groups in all measures of physical function. Given the paucity of studies, no subgroup analyses were conducted.

#### Adherence

Adherence to interventions were between 30% and 96%. Most studies reported medium to high adherence via self-reported daily logs [49–52, 54, 55, 58]. Adherence rates decreased over time in two studies, from 40% at month-6 to 30% at month-24 [56], and from 93 to 60% over 6 months [49] respectively. Amongst all studies, six involved motivational calls which might affect the intervention adherence. Specifically, the study [49] which reported adherence had decreased by time, only had motivational calls in the first 2 months. One study [57] did not report adherence. Given the heterogeneity of methods and thresholds for defining adherence, and data availability, a meta-regression for determining the impact of adherence on effectiveness was not performed.

#### Adverse events

Six out of ten trials reported adverse event data, involving only 45 participants. In particular, five adverse events (falls) related to the intervention, which led to minor injuries, were reported in three participants in the StandingTall programme [56]; four adverse events, associated with participants’ pre-existing knee pain, were reported in the FlexToBa programme [49]; and few adverse events (i.e., leg pain, joint pain, and breathlessness), which were not limiting the programme execution, were reported in the EXCITE programme [53]. No adverse events related to the interventions were reported in other interventions [52, 54, 57]. Overall, no serious adverse events relating to the interventions were reported in the included studies.

### Methodological quality

**Fig. 7:**
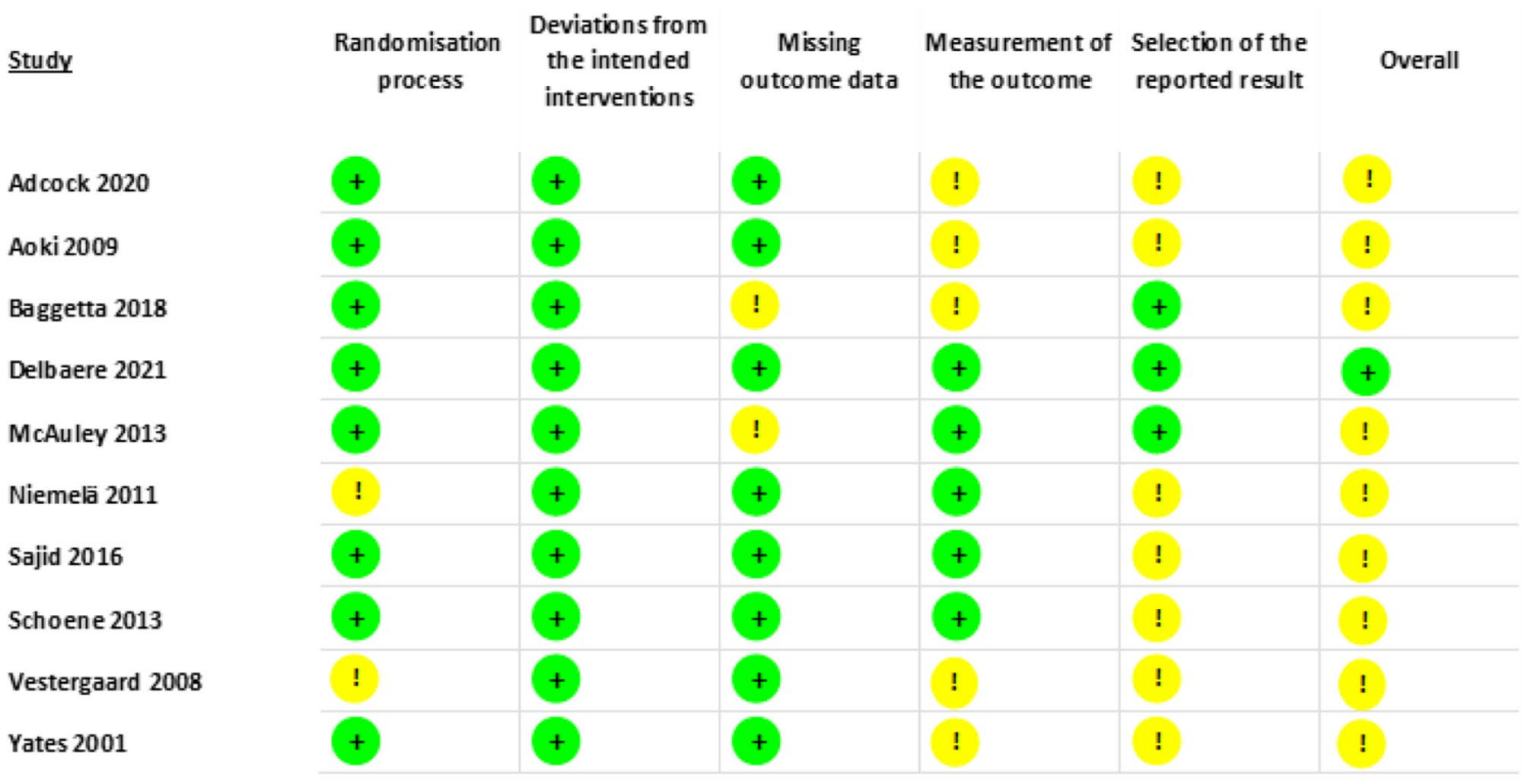
Risk of bias assessment for included studies where green = low risk of bias, yellow = moderate risk of bias, and red = high risk of bias

All 10 of the included studies were assessed for risk of bias (Fig. 7). One study was considered low risk of bias for all domains [56] and the remaining nine studies had at least one domain judged to be moderate risk of bias [49–55, 57, 58]. Particularly, half of the studies reported that investigators were aware of allocation where the investigator-reported outcomes may involve some judgement, with regards to assessor blinding. Overall, nine studies were considered to be of moderate risk of bias with moderate quality and one was judged as low risk of bias with high quality.

### Quality of evidence

The GRADE assessment [45] revealed that the overall certainty of evidence for the effectiveness of unsupervised home-based exercise interventions ranges from low-to-moderate (Table 1). Specifically, the evidence for the 5 x sit-to-stand time was rated as low due to moderate risk of bias and very serious concerns with imprecision. For knee extension strength, the evidence was rated as very low because of moderate risk of bias, very serious imprecision, and high inconsistency. Evidence for 10 m walking speed and the TUG was rated as moderate, reflecting moderate risk of bias with serious imprecision. The evidence for SPPB was rated as very low due to very serious imprecision and high inconsistency.

**Table 1:** GRADE assessment of evidence quality

Outcome	No. of Studies	Study Design	Relative Effect	Confidence Interval	Risk of Bias	Inconsistency	Indirectness	Imprecision	Quality of Evidence
5 x time sit-to-stand	5	RCTs	-0.18	[-0.36, 0.00]	Moderate	Not serious (I²=0%)	Not serious	Very serious	⊕⊕◯◯ Low
Knee extension strength	2	RCTs	0.21	[-0.60, 1.03]	Moderate	Very serious (I²=71%)	Not serious	Very serious	⊕◯◯◯ Very low
10 m walking speed	4	RCTs	0.18	[-0.10, 0.46]	Moderate	Not serious (I²=30%)	Not serious	Serious	⊕⊕⊕◯ Moderate
TUG	2	RCTs	-0.08	[-0.32, 0.17]	Moderate	Not serious (I²=0%)	Not serious	Serious	⊕⊕⊕◯ Moderate
SPPB	3	RCTs	1.14	[-1.09, 3.37]	Moderate	Very serious (I²=98%)	Not serious	Very serious	⊕◯◯◯ Very low

RCT = Randomised controlled trial; TUG = Timed up and go test; SPPB = Short Physical Performance Battery

## Discussion

This systematic review and meta-analysis aimed to explore the effect of unsupervised home-based exercises on lower extremity physical function in older adults, and whether the effects varied across Western and Eastern cultures. However, no Mandarin articles met the inclusion criteria for this review. The absence of Mandarin studies highlights a significant research gap in understanding of the effectiveness of unsupervised home-based exercises in diverse cultural contexts. Nonetheless, the available evidence from Western databases indicated that completely unsupervised home-based exercise was not effective for improving lower limb function in older adults.

The multifaceted advantages of home-based exercise programmes may overcome barriers to exercise due to execution convenience and low cost [35, 61]. However, the meta-analysis of included studies indicated that entirely unsupervised home-based exercise interventions did not significantly improve 5 x sit-to-stand time, knee extension strength, 10 m walking speed, TUG performance, or SPPB score in older adults. Eight studies (839 participants) were used for statistical meta-analyses rendering the study and participant sample sizes small. The GRADE assessment highlighted significant variability in the certainty of evidence across the studies and measures. The certainty of evidence for knee extension strength and SPPB was considered very low due to substantial imprecision and inconsistency and was considered low for the 5 x sit-to-stand test due to concerns related to imprecision. The 10 m walk and TUG were had a moderate certainty of evidence. There was also a high statistical heterogeneity observed in the analysis of SPPB and knee extension strength, which may limit the reliability of the meta-analysis for these particular outcomes [62]. However, for other outcomes, the heterogeneity was not as pronounced. When reflecting on the quality of the available evidence, the present meta-analysis may not fully capture the potential effects of unsupervised home-based exercise interventions [63].

The common excluding reasons were interventions with supervision or home visits, and active control groups, with 41% and 24% of potential English and Mandarin excluded due to interventions with supervision respectively. As such, relatively few randomised controlled trials of unsupervised, home-based, exercise interventions with usual care control arms for older adults have been evaluated. The small number of high-quality intervention trials is somewhat surprising given that supervision of home-based exercise can be a resource intensive component of trial delivery. Moreover, in research aiming to produce scalable, low-cost exercise programmes for large numbers of potential users, evidence that the programme can be effective without supervision would seem to be a prerequisite.

Whilst the present study reports a lack of effectiveness for completely unsupervised homebased exercise, previous literature suggests that even minimal supervision may be an important component of exercise interventions for older adults. Mahjur and Norasteh [38] reviewed the effectiveness of minimally to none supervised (i.e., supervision ratios lower than 33%) home-based exercises in people over 60 and found significant intervention effect in TUG and Berg balance scale. Similarly, a meta-analysis showed that home-based exercises with supervision ratios less than 20% still led to significant improvements in balance and muscle functions in healthy older adults [39]. Another systematic review indicated that even supervision ratios lower than 15% for home-based exercise programmes had positive effects on lower body strength and mobility in people over 60 [37]. These studies suggest that home-based exercise per se does not lack evidence of effectiveness, however some degree of supervision may be required. Further research could address why unsupervised interventions specifically did not induce significant improvements in lower limb function and whether different types of supervision could affect outcomes.

Other methodological issues such as sample size, statistical power, lack of robustness in study designs, and inter-study heterogeneity (as seen in the present SPPB and knee extension data) may bias the results when conducting meta-analyses [64–66]. The present study had fewer studies and participants that previous similar meta-analyses. These earlier meta-analyses incorporated 12 to 17 studies, involving a larger participant pool ranging from 1160 to 2570 participants [37–39, 67], whereas the present analysis comprised 8 trials with 839 participants. However, trial duration was similar between studies included in previous and the present meta-analysis. The trials included in the present study lasted 4 weeks to 24 months with participants engaging in exercise on average three times per week for 10 to 40 min per session, with medium to high adherence This aligns closely with similar studies where intervention durations ranged from 4 weeks to 26 months, with sessions occurring on average three times per week involving 10 to 60 min each. This may suggest that the specific exercises or intensity of the exercises differed between unsupervised and minimally supervised interventions. Alternatively, ceiling effects in healthy and high-functioning participants [52, 57], and some control groups receiving healthy ageing advice which might induce behaviour change may have affected the outcomes and reduce the statistical power [53, 56]. These original study design considerations may have led to lower effect sizes in the present meta-analysis compared supervised home-based exercise [38].

Importantly, only 5% of participants (45 out of 907 participants) reported adverse events during the interventions, and none were classified as severe (or serious) adverse events. Of all reported adverse events, few were related to the interventions. These intervention-induced adverse events included falls, muscle soreness, joint pain, and shortness of breath. This may confirm previous research demonstrating that unsupervised home-based exercise interventions are safe and feasible for older adults. Indeed, the safety of the leisure setting exercise for older adults is undoubtedly important to consider in light of the potential risks in performing exercises [68, 69]. The exercise types and intensities need to be considered when designing home-based exercise programmes.

This is the first systematic review and meta-analysis that integrated data from randomised control trials on the effectiveness of unsupervised home-based exercise programmes on lower extremity functions in older adults. Additionally, most prior reviews were limited to the monolingual scope of searches, and this current study to systematically search not only English literature but also Mandarin literature, although no eligible Mandarin studies were identified. The strength of this review lies in its comprehensive approach to synthesizing available evidence; however, the low number of eligible studies and overall small sample sizes weaken the study quality due to underpowered analyses. Furthermore, the lack of consistent outcome measures in the studies used to run the meta-analysis in a single outcome measure may mislead the findings and overlook the generalizability, and two otherwise eligible articles did not report sufficient data to be included in the meta-analysis. The moderate to high heterogeneity among the included studies for SPPB and knee extensor data is another limitation of this meta-analysis and may reduce the accuracy of the results. Moreover, it was not possible to analyze differences between healthy versus non-healthy individuals, length of intervention, or exercise modality, due to the low number of included studies and participants.

## Conclusions

This systematic review and meta-analysis demonstrated that unsupervised home-based exercise programmes did not significantly improve in lower extremity physical functions, including muscle strength, balance, and gait speed, in older adults compared to control conditions. While the included studies did not present major concerns regarding risk of bias, the limited data available, methodological variability, and observed statistical heterogeneity, particularly in knee extension strength and SPPB, should be considered when interpreting these results. Given these findings, strategies to support home-based exercise interventions aiming to improve physical function in older adults are warranted to maximise effectiveness.

## Electronic supplementary material

Below is the link to the electronic supplementary material.

Supplementary Material 1

## Data Availability

The analytic methods used in this research are available upon request from the first or corresponding authors.

## Abbreviations

RCT: Randomised Controlled Trial
SPPB: Short Physical Performance Battery
PROSPERO: International Prospective Register of Systematic Reviews
PICOS: Population, Intervention, Comparison, Outcomes, and Study
TUG: Time-Up-to-Go
