# Supplementary material for: The effectiveness of unsupervised home-based exercise for improving lower extremity physical function in older adults in Western and Eastern cultures: a systematic review and meta-analysis
Source: BMC Geriatr. 2024 Oct 1;24:800. doi: 10.1186/s12877-024-05393-4 (PMC11443890; doi:10.1186/s12877-024-05393-4)
Supplement: Supplementary file 1 — Supplementary Material 1 [file 12877_2024_5393_MOESM1_ESM.docx]

**The effectiveness of unsupervised home-based exercise for improving lower extremity physical function in older adults in Western and Eastern cultures: a systematic review and meta-analysis**

**Supplementary A. PRISMA checklist**

| **Section and Topic** | **Item #** | **Checklist item** | **Location where item is reported** |
| --- | --- | --- | --- |
| **TITLE** | | |  |
| Title | 1 | Identify the report as a systematic review. | Title page |
| **ABSTRACT** | | |  |
| Abstract | 2 | See the PRISMA 2020 for Abstracts checklist. | 1 |
| **INTRODUCTION** | | |  |
| Rationale | 3 | Describe the rationale for the review in the context of existing knowledge. | 2-3 |
| Objectives | 4 | Provide an explicit statement of the objective(s) or question(s) the review addresses. | 3 |
| **METHODS** | | |  |
| Eligibility criteria | 5 | Specify the inclusion and exclusion criteria for the review and how studies were grouped for the syntheses. | 4 |
| Information sources | 6 | Specify all databases, registers, websites, organisations, reference lists and other sources searched or consulted to identify studies. Specify the date when each source was last searched or consulted. | 4 and Supplementary file B |
| Search strategy | 7 | Present the full search strategies for all databases, registers and websites, including any filters and limits used. | 4 and Supplementary file B |
| Selection process | 8 | Specify the methods used to decide whether a study met the inclusion criteria of the review, including how many reviewers screened each record and each report retrieved, whether they worked independently, and if applicable, details of automation tools used in the process. | 5 |
| Data collection process | 9 | Specify the methods used to collect data from reports, including how many reviewers collected data from each report, whether they worked independently, any processes for obtaining or confirming data from study investigators, and if applicable, details of automation tools used in the process. | 5 |
| Data items | 10a | List and define all outcomes for which data were sought. Specify whether all results that were compatible with each outcome domain in each study were sought (e.g. for all measures, time points, analyses), and if not, the methods used to decide which results to collect. | 3-5 |
|  | 10b | List and define all other variables for which data were sought (e.g. participant and intervention characteristics, funding sources). Describe any assumptions made about any missing or unclear information. | 3-5 |
| Study risk of bias assessment | 11 | Specify the methods used to assess risk of bias in the included studies, including details of the tool(s) used, how many reviewers assessed each study and whether they worked independently, and if applicable, details of automation tools used in the process. | 5 |
| Effect measures | 12 | Specify for each outcome the effect measure(s) (e.g. risk ratio, mean difference) used in the synthesis or presentation of results. | 5 |
| Synthesis methods | 13a | Describe the processes used to decide which studies were eligible for each synthesis (e.g. tabulating the study intervention characteristics and comparing against the planned groups for each synthesis (item #5)). | 5 |
|  | 13b | Describe any methods required to prepare the data for presentation or synthesis, such as handling of missing summary statistics, or data conversions. | 5 |
|  | 13c | Describe any methods used to tabulate or visually display results of individual studies and syntheses. | 5 |
|  | 13d | Describe any methods used to synthesize results and provide a rationale for the choice(s). If meta-analysis was performed, describe the model(s), method(s) to identify the presence and extent of statistical heterogeneity, and software package(s) used. | 5 |
|  | 13e | Describe any methods used to explore possible causes of heterogeneity among study results (e.g. subgroup analysis, meta-regression). | 5 |
|  | 13f | Describe any sensitivity analyses conducted to assess robustness of the synthesized results. | - |
| Reporting bias assessment | 14 | Describe any methods used to assess risk of bias due to missing results in a synthesis (arising from reporting biases). | 5 |
| Certainty assessment | 15 | Describe any methods used to assess certainty (or confidence) in the body of evidence for an outcome. | 5 |
| **RESULTS** | | |  |
| Study selection | 16a | Describe the results of the search and selection process, from the number of records identified in the search to the number of studies included in the review, ideally using a flow diagram. | 6 |
|  | 16b | Cite studies that might appear to meet the inclusion criteria, but which were excluded, and explain why they were excluded. | 6 and Figure 1 |
| Study characteristics | 17 | Cite each included study and present its characteristics. | 6 and Supplementary file C |
| Risk of bias in studies | 18 | Present assessments of risk of bias for each included study. | 9 |
| Results of individual studies | 19 | For all outcomes, present, for each study: (a) summary statistics for each group (where appropriate) and (b) an effect estimate and its precision (e.g. confidence/credible interval), ideally using structured tables or plots. | 6-9 |
| Results of syntheses | 20a | For each synthesis, briefly summarise the characteristics and risk of bias among contributing studies. | 7-9 |
|  | 20b | Present results of all statistical syntheses conducted. If meta-analysis was done, present for each the summary estimate and its precision (e.g. confidence/credible interval) and measures of statistical heterogeneity. If comparing groups, describe the direction of the effect. | 7-8 and Figure 2-6 |
|  | 20c | Present results of all investigations of possible causes of heterogeneity among study results. | 7-8 and Figure 2-6 |
|  | 20d | Present results of all sensitivity analyses conducted to assess the robustness of the synthesized results. | - |
| Reporting biases | 21 | Present assessments of risk of bias due to missing results (arising from reporting biases) for each synthesis assessed. | 9 |
| Certainty of evidence | 22 | Present assessments of certainty (or confidence) in the body of evidence for each outcome assessed. | 9 |
| **DISCUSSION** | | |  |
| Discussion | 23a | Provide a general interpretation of the results in the context of other evidence. | 9-12 |
|  | 23b | Discuss any limitations of the evidence included in the review. | 12 |
|  | 23c | Discuss any limitations of the review processes used. | 12 |
|  | 23d | Discuss implications of the results for practice, policy, and future research. | 13 |
| **OTHER INFORMATION** | | |  |
| Registration and protocol | 24a | Provide registration information for the review, including register name and registration number, or state that the review was not registered. | 3-4 |
|  | 24b | Indicate where the review protocol can be accessed, or state that a protocol was not prepared. | 3-4 |
|  | 24c | Describe and explain any amendments to information provided at registration or in the protocol. | - |
| Support | 25 | Describe sources of financial or non-financial support for the review, and the role of the funders or sponsors in the review. | 13 |
| Competing interests | 26 | Declare any competing interests of review authors. | 13 |
| Availability of data, code and other materials | 27 | Report which of the following are publicly available and where they can be found: template data collection forms; data extracted from included studies; data used for all analyses; analytic code; any other materials used in the review. | - |

*From:*  Page MJ, McKenzie JE, Bossuyt PM, Boutron I, Hoffmann TC, Mulrow CD, et al. The PRISMA 2020 statement: an updated guideline for reporting systematic reviews. BMJ 2021;372:n71. doi: 10.1136/bmj.n71

**Supplementary B. Searching strategies and terms**

**English search strategy and terms**

The search was built around five groups of key words: Population (e.g. ‘’older’’, ‘’retired’’, “over 65*”, ‘’adults’’, ‘’older adults’’); Intervention (e.g. ‘’physical activity’’, ‘’strength training’’, ‘’balance training’’, ‘’yoga’’, ‘’otago’’, ‘’pilates’’, ‘’ tai chi’’, ‘’personal training’’); Context (e.g. ‘’home based’’, ‘’residen*’’, ‘’habitation’’, ‘’residence’’); Study design (e.g. ‘’RCT’’, ‘’rct’’, ‘’randomi?ed controlled trial’’); and Outcome (e.g. ‘’ physical function’’, ‘’strength’’, ‘’balance’’, ‘’lower extremity function’’, ‘’mobility’’).

*-Example of search terms used in Pubmed publication database*

((elder* OR elderly OR older OR retire* OR retired OR "over 65*" OR "over sixty five*" OR senior* OR senior OR pensioner* OR pensioners OR age* OR aged OR layer li*'' OR adult* OR adults OR later life)) AND ("physical* activ*" OR physical activity OR "exercise participation" OR strength and balance training OR strength training OR balance training OR resistan* OR resistance OR exercise OR exercise* OR yoga OR otago OR keep fit OR physiotherap* OR cal?isthen* OR isometric* OR isometric OR isotonic OR work?out OR rehab* OR rehabilitation OR circuit training OR plates OR tai chi*'' OR tai chi OR tai chi chung OR qigong OR weightlift* OR weightlifting OR fitness OR function* OR function OR personal training OR PT OR aerobic* OR aerobic OR sport OR movement OR step OR climbing)) AND (home based OR home-based OR home OR residen* OR abode OR habituation OR residence)) AND (rct OR RCT OR "randomi?ed controlled trial*" OR "randomi?ed controlled trial")) AND (physical function*'' OR physical function OR strength OR balance OR lower extremity function*'' OR lower leg function*'' OR fall* OR falling OR falls OR mobilit* OR mobility OR power OR frail* OR frail OR frailty OR ‘’daily active*'' OR daily activity OR acceptability OR preference OR adherence OR maintenance OR belie* OR attitud* OR believe OR attitude))

**Mandarin search strategy and terms**

The search for the Mandarin literature was built with five groups of key words: Population (e.g. ‘’老人’’, ‘’退休’’, “65歲”, ‘’成人’’, ‘’銀髮族’’, ‘’老齡人口’’); Intervention (e.g. ‘’身體活動’’, ‘’重量訓練’’, ‘’平衡訓練’’, ‘’瑜珈’’, ‘’奧塔格運動’’, ‘’皮拉提斯’’, ‘’太極拳’’, ‘’自主訓練’’); Context (e.g. ‘’居家’’, ‘’自主’’, ‘’住所’’, ‘’住宅’’); Study design (e.g. ‘’RCT’’, ‘’隨機對照試驗’’, ‘’隨機分配’’); and Outcome (e.g. ‘’ 身體功能’’, ‘’肌力’’, ‘’平衡’’, ‘’下肢功能’’, ‘’活動度’’).

See the below table for the searching terms in Mandarin:

| **Population** |  | **Intervention** |  | **Context** |  | **Study Design** |  | **Outcome** |  |
| --- | --- | --- | --- | --- | --- | --- | --- | --- | --- |
| Older Adults | 老人 | Exercise | 運動 | Home based | 居家 | RCT |  | Strength | 力量/肌力 |
| Elderly | 銀髮族 | Strength training | 重量訓練/力量訓練/體能訓練 | Home | 家庭 | Randomised Controlled Trial | 隨機對照試驗/隨機對照研究/隨機分配/隨機分派/隨機控制 | Balance | 平衡 |
| Elders | 老年人/老年群體 | Balance training | 平衡/平衡訓練/平衡運動 | At home | 在家 |  |  | Physical function | 身體功能/身体机能/躯体功能 |
| Senior citizen | 高齡族群/高齡人群/高龄群体/长者 | Resistance | 阻力 | Personal | 自主 |  |  | Mobility | 活動度/柔韌性/灵活度 |
| Adults | 成人/成年人 | Training | 訓練/鍛鍊 | Residence | 宅/住宅 |  |  | Lower leg function | 下肢功能 |
| Over 65 | 65歲以上 | Fitness | 體適能/體能 | Abode | 居住/舍 |  |  | Power | 爆發力 |
| 65+ |  | Function | 功能/功能性 | Habitation | 住所 |  |  | Activities of daily life | 身體活動/日常身體活動/日常锻炼/日常活动 |
| Pensioners | 養老/退休人士/退休人员/养老金领取者 | Tai Chi | 太極/太極拳 |  |  |  |  |  |  |
| Ageing | 銀髮族/老龄人口 | Yoga | 瑜珈/瑜伽 |  |  |  |  | Acceptability | 接受度/接受性 |
| Later life | 晚年 | Otago | 奧塔格運動 |  |  |  |  | Preference | 偏好/偏愛 |
| Retired | 退休 | Activity | 活動 |  |  |  |  | Adherence | 依從姓/運動遵從度/堅持度 |
|  |  | Physical activity | 身體活動 |  |  |  |  | Maintenance | 持續性 |
|  |  | Personal training | 私人訓練/一對一訓練/自主訓練/自主運動訓練 |  |  |  |  |  |  |
|  |  | Keep-fit | 維持體適能/保持健康/保持身材 |  |  |  |  |  |  |
|  |  | Aerobics | 有氧/有氧運動 |  |  |  |  |  |  |
|  |  | Circuit training | 循環式訓練/循环训练 |  |  |  |  |  |  |
|  |  | Sport | 運動/體育 |  |  |  |  |  |  |
|  |  | movement | 動作 |  |  |  |  |  |  |
|  |  | Physiotherapy | 復健/治療/理疗 |  |  |  |  |  |  |
|  |  | Callisthenics | 體操/健美操/廣場舞/有氧舞蹈 |  |  |  |  |  |  |
|  |  | Isometrics | 等長訓練/等長收縮訓練/静力训练/静力性训练 |  |  |  |  |  |  |
|  |  | Isotonic | 等張訓練 |  |  |  |  |  |  |
|  |  | Work out | 锻炼 |  |  |  |  |  |  |
|  |  | rehabilitation | 修復/復健/康复 |  |  |  |  |  |  |
|  |  | step | 階梯/台阶 |  |  |  |  |  |  |
|  |  | climbing | 登階 |  |  |  |  |  |  |
|  |  | pilates | 皮拉提斯/普拉提 |  |  |  |  |  |  |
|  |  | qigong | 氣功 |  |  |  |  |  |  |
|  |  | Weightlifting | 举重 |  |  |  |  |  |  |

**Supplementary C. Summary of included studies**

| **Author, date, setting, location** | **Population/health condition, age, gender** | **Intervention: type, delivery type, duration, frequency, length, additional support** | **Lower extremity outcome measures** | **Adherence and record methods** |
| --- | --- | --- | --- | --- |
| Adcock 2020 (Adcock et al., 2020)  Home-based exercise intervention, Switzerland. | Healthy older adults, able to stand at least for 10 minutes without assistance.  I: n = 15, 77 yrs.  67% female  C: n = 16, 70 yrs.  38% female | I: Tai Chi-inspired exercises, dancing, and step-based cognitive games, utilising computer software and hardware (i.e., straps).  C: Usual care (normal daily living).  18 weeks, 3 times/week, 30-40 minutes/session.  One-off 1-hour introductory session, and biweekly motivational call. | SPPB extended balance test, 30-sec sit-to-stand, 2min stepping test, and 10m walking speed. | 70% of the  total training sessions, daily log sheets |
| Aoki 2009 (Aoki et al., 2009)  Home-based exercise intervention, Tokyo, Japan. | Female older adults with knee osteoarthritis.  I: n = 17, 72.3 yrs.  C: n = 19, 74.4 yrs. | I: Knee stretching exercises, using written instructions.  C: Usual care (normal daily living).  30-140 days depends on the time of total knee arthroplasty, 7 times/week, 30 seconds for each exercise for 10 reps. | 10m gait speed, knee ROM in supine position, and knee ROM from maximum flexion to extension during gait (with a goniometer) | 93% of the total training sessions, daily log sheets |
| Baggetta 2018 (Baggetta et al., 2018)  Home-based exercise intervention, Reggio Calabria, Italy. | Older adults with dialysis, able to walk 550m in 6 min.  I: n = 53, 73 yrs.  36% female  C: n = 62, 75 yrs.  34% female | I: Personalised home-based walking exercise, using online video instructions (i.e., YouTube).  C: Usual care with healthy ageing advice.  6 months, 3 times/week, 15 minutes/session.  5 motivational calls during the intervention. | 6-min walking distance and 5-time sit-to-stand test | 49% had low adherence and 51% had high adherence (undefined adherence definition), assessed by evaluating the residual battery charge in the metronome |
| Delbaere 2021 (Delbaere et al., 2021)  Home-based exercise intervention, Sydney metropolitan area, Australia. | Healthy and independent-living community dwelling older adults, able to walk household distances without the use of a walking aid.  I: n = 114, 77.1 yrs.  70% female  C: n = 112, 77.7 yrs.  65% female | I: The StandingTall programme focusing on standing balance, targeted stepping, and step-up exercises, with exercise equipment (foam cushion, stepping box, exercise mat), utilising tablet computers.  C: Usual care with health education.  2 years, started from 40mins/week increased to 120mins/week by week 9.  One-off 1-hour introductory session. | Standing balance, maximum forward-backwards balance, and controlled leaning balance, time up-to-go, SPPB, and 10m walking speed. | 40%, 34%, 33%, and 30% of participants achieved the prescribed dose over 6, 12, 18, and 24 months respectively, automatic data from tablet computers. |
| McAuley 2013 (McAuley et al., 2013)  Home-based DVD exercise intervention, east-central Illinois, USA. | Physically inactive older adults.  I: n = 158, 70.6 yrs.  72% female  C: n = 149, 71.4 yrs.  83% female | I: Six progressive exercise sessions focusing on balance, strength, and flexibility with exercise equipment (two resistance bands and a yoga mat), following Flexibility, Toning, and Balance (FlexToBa) trial, using DVD delivery.  C: Usual care with healthy ageing advice.  6 months, 3 times/week, 6-month follow-up.  Biweekly motivational call in the first 2 months. | SPPB and sit-and-reach test. | 76% (range: 93% at month 1 to 60% at month 6) of the  total training sessions, daily log sheets |
| Niemelä 2011 (Niemelä et al., 2011)  Home-based exercise intervention, Finland. | Community dwelling female older adults.  I: n = 26, 79.8 yrs.  C: n = 25, 80.7 yrs. | I: Ten rocking-chair exercises focusing on improving muscle strength in the lower limbs and mobility, using written instructions.  C: Usual care (normal daily living).  6 weeks, 10 times/week, 15 minutes/session.  One-off 1-hour introductory session. | Maximal isometric knee extension, 5-time sit-to-stand, stand on one leg test, the Berg Balance Scale, and 10m walking speed. | 96% of the total training sessions, daily log sheets |
| Sajid 2016 (Sajid et al., 2016)  Home-based exercise intervention, Chicago (Illinois) and New York, USA. | Sedentary male older adults with prostate cancer on androgen deprivation therapy, able to walk 4m.  I1: n = 8, 70-87 yrs.  I2: n = 6, 67-93 yrs.  C: n = 5, 67-80 yrs. | I1: A tailored, multi-component technology-mediated exercise program utilising the Wii-Fit technology.  I2: A tailored, multi-component, home-based aerobic and progressive resistance exercise program (including moderately intense aerobic walking exercise and low to moderate intensity progressive resistance exercises), using written instructions.  C: Usual care (normal daily living).  6 weeks, 5 times/week, 12-week follow-up.  One-off 45-min introductory session, and weekly motivational call. | SPPB. | 70% (undefined adherence definition), daily log sheets |
| Schoene 2013 (Schoene et al., 2013)  Home-based videogame exercise intervention, retirement village in Sydney, Australia. | Healthy and independent-living older adults, able to walk without a walking aid for 20 m and to step in place unassisted on a step pad.  I: n = 15, 77.5 yrs.  C: n = 17, 78.4 yrs. | I: Step video games with three levels of difficulty through a computerised step pad system (modified Dance Dance Revolution game, StepMania), utilising the computer unit.  C: Usual care (normal daily living).  8 weeks, 2-3 times/week, 10-20 minutes/session.  One-off 90-min introductory session, and 4 motivational calls. | Time up-to go, 5-time sit-to-stand, alternative step test for functional balance, and the Physiological Profile Assessment (including isometric knee extension, anterior-post Sway, and med-lat Sway tests). | N/A |
| Vestergaard 2008 (Vestergaard et al., 2008)  Home-based DVD exercise intervention, Denmark. | Physically inactive female older adults.  I: n = 25, 81 yrs.  C: n = 28, 82.7 yrs. | I: Home-based exercise comprised 15 minutes of warm-up, focusing on flexibility and dynamic balance exercises, 6 minutes of strengthening exercises using the elastic band for arms and legs, and 5 minutes of aerobic exercises, using DVD delivery.  C: Usual care (normal daily living).  5 months, 3 times/week, 26 minutes/session.  One-off 1-hour introductory session, and biweekly motivational call. | Maximal leg extensor power, 5-time sit-to-stand, semi-tandem stand balance test, the Physical Performance Test for assessing activities of daily living, and 10m walking speed. | 89.2% (ranging from 45-100%) (undefined adherence definition), daily log sheets |
| Yates 2001 (Yates & Dunnagan, 2001)  Home-based exercise intervention, rural southwest Montana, USA. | Independent-living community dwelling older adults.  I: n = 18, 69-90 yrs.  72% female  C: n = 19, 69-88 yrs.  68% female | I: 19 chair-based exercises for improving strength (with 5-lb adjustable weights), coordination, balance, and mobility, following guidelines of the Movement Matters: Home Based Exercise Program, using written instructions.  C: Usual care (normal daily living).  10 weeks, 3 times/week, 15 minutes/session.  One-off 1-hour introductory session, and health education. | Time up-to-go, Tinetti balance test, and lower extremity power. | 55% of participants achieved the prescribed dose, daily log sheets |
| I= intervention group, C= control group, yrs.= years, SPPB= short physical performance battery test, ROM= range of motion. | | | | |
